# Rainwater as a Source of Drinking Water: Health Impacts and Rainwater Treatment

**DOI:** 10.1155/2019/1760950

**Published:** 2019-07-11

**Authors:** Khayan Khayan, Adi Heru Husodo, Indwiani Astuti, Sudarmadji Sudarmadji, Tjut Sugandawaty Djohan

**Affiliations:** ^1^Department of Environmental Health, Poltekkes Kemenkes, Pontianak, Indonesia; ^2^Medicine Faculty, Universitas of Gadjah Mada, Yogyakarta, Indonesia; ^3^Geography Faculty, Universitas of Gadjah Mada, Yogyakarta, Indonesia; ^4^Laboratory of Ecology and Conservation, Biology, Faculty, Universitas of Gadjah Mada, Yogyakarta 55281, Indonesia

## Abstract

Rainwater is the main source of drinking water in tropical communities, especially in West Kalimantan. Air contamination causes rainwater to become acidic and cloudy and adds heavy metals such as Pb into rainwater. In addition to pollution, the way in which the rainwater is collected such as through zinc roofing also exposes the rainwater to heavy metals. The presence of Pb in rainwater will have an impact on the health of the community in the long run. The model of simple water treatment using filtration is needed to overcome this problem with the use of media available in the region. The media used are in the form of mollusk sand and activated carbon. In the end, the mollusk sand filtration model and activated carbon sorption were effectively used to filter polluted rainwater to be safe for consumption.

## 1. Introduction

The amount of surface water and groundwater in West Kalimantan is abundant, but with respect to quality, it does not meet the requirements of being used as drinking water [1, 2]. Surface water such as rivers, lakes, and reservoirs has a high turbidity level, and Fe content in groundwater exceeds the threshold value, triggering the usage of rainwater as a source of drinking water [3]. Rainwater is used as a source of drinking water not only in West Kalimantan, Indonesia, but also in other tropical regions, such as Australia and Africa [4, 5].

Increasing population and industrial growth have an impact on air quality [6]. As in West Kalimantan, besides the industrialization factor, the condition of peatlands is a contributor to air pollution due to fires in the dry season [7]. Under highly polluted air conditions, the quality of rainwater normally gets affected, for example, increase in the heavy metal content, such as lead (Pb) [8, 9].

Before being collected in a water tank, the rainwater will fall on the tin roof used by the community as the roof of the house. In the process of making zinc roofs, each part of the roof is added or coated with Pb. Pb serves to strengthen the zinc layer bond with the iron layer [10–12]. Lead is also used to prevent corrosion of the zinc roof. In Pontianak City, it is recognized that rainwater is corrosive because it has aggressive CO_2_ and low pH in the range of 4.78–5.85 [13]. So that the tin roof is easily corroded and Pb as a roof coating also gets dissolved and added into rainwater [14].

Temporal demand for studying the effects of drinking water exposed to lead is attractive because of its toxicological effects, such as kidney disease, cancer, and cognitive impairment from chronic exposure in the short term [15, 16]. From several studies, the effects of long-term lead exposure have been linked to various forms of cancer, nephrotoxicity, central nervous system effects, and cardiovascular disease in humans. Other effects of Pb exposure cause enzyme disorders, anemia, mental disorders, and hyperactivity in children, underweight and premature conditions in newborns, and increased high blood pressure in adults [10, 11]. In our body, Pb accumulates in the bone in the long term, around 20–30 years, and becomes chronic [11].

Measurement of Pb exposure levels in rainwater as a source of drinking water needs to be done as an assessment of health risks by exposure to this heavy metal. Measurement of short-term Pb health effects can be done by measuring Pb levels in urine. Our goal is to determine the level of rainwater exposure to Pb causing health risks and bring about efforts to use rainwater as a safe source of drinking water by applying appropriate technology using media from activated carbon and mollusk sand.

## 2. Materials and Methods

### 2.1. Study Area and Design

**Figure 1:**
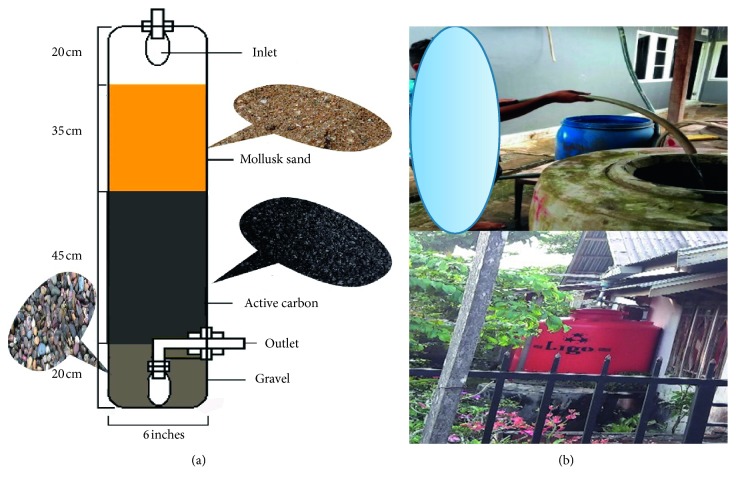
(a) Filtration device with mollusk sand and activated carbon media. (b) The way people collect rainwater.

**Figure 2:**
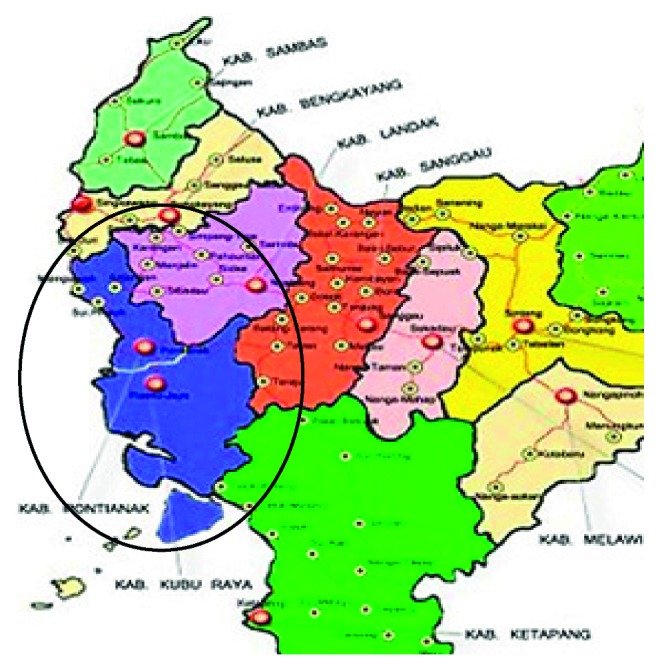
The location of the study conducted.

The study was a cross-sectional and experimental study. This cross-sectional study was carried out to analyze the effect of presence of Pb in urine, while the experimental one was used for testing rainwater treatment devices designed to reduce the content of lead (Pb) and turbidity and increase the pH of rainwater. The treatment is performed using appropriate technology to reduce exposure of rainwater to lead (Pb) through filtering shellfish sand and absorption with activated carbon in a filter tube (Figure 1). Rainwater samples from two target areas in West Kalimantan, Indonesia, were collected to represent urban and rural areas, namely, Pontianak City and Kubu Raya District (Figure 2). In the city, two target locations were chosen, namely, Siantan Hulu and Central Siantan Districts, while in Kubu Raya District, the locations of Limbung village and Arang Limbung village were chosen. This research was conducted for 12 months from January to December 2016.

### 2.2. Sample Size and Sampling

The sample comes from a group of houses that hold rainwater through a zinc roof as a source of drinking water. A total of 40 houses were sampled in this study. The sampling technique uses simple random clustering with the determination of the sampling done in stages and dividing it into several clusters. The sample area is represented by two target regions. The city is represented by the subdistricts of North Pontianak, and rural area is represented by Sungai Raya District. Rainwater samples were taken at intervals of 0–20 minutes as much as 2.5 liters of rain. Rainwater samples were taken to meet laboratory requirements, while urine samples were collected from the residents who lived the longest at home with the highest consumption of rainwater.

### 2.3. Sampling Technique

Water was poured into a 2.5 mL polyurethane sample bottle during shipment of the sample, tightly closed, and properly labeled to prevent cross contamination. Samples were sent to the laboratory in the form of water samples before processing, and the results of processing using shellfish sand and activated carbon are given in Figure 1. Water parameters such as pH and turbidity were taken in the field. Samples taken immediately may be brought to the laboratory for testing by inserting into a box containing ice. The samples were collected and processed every time it rained in the area that had been used as the research target. Urine samples were taken from residents of the house who mostly stayed at home and had the highest consumption of rainwater. Urine sample was taken in the morning after wake up. A urine sample was taken to determine the health effects associated with public health problems with a theoretical review.

### 2.4. Characteristics of Filtration Media

Activated carbon has diverse surface characteristics and pore size distribution, and the characteristics of this activated carbon play an important role in the adsorption of contaminants. Activated carbon used in research is granular or irregular with a size of 0.2–5 mm. Activated carbon is obtained from coconut shell. The manufacturing process starts with the dehydration, the process of removing the water content from the raw material by heating it in an oven at a temperature of 170°C. At a temperature of around 275°C, carbon decomposition takes place and tar, methanol, phenol, and other by-products are formed. Nearly 80% of carbon is obtained at temperatures of 400–600°C. Charcoal was formed from carbonization at a temperature of more than 400°C, and activation was carried out chemically using calcium chloride (CaCl_2_) [17, 18]. The mollusk sand used comes from the coastal area of West Kalimantan. Mollusk sand is the result of weathering the shell of the shellfish. Mollusk sand used is 0.2–5 mm in size.

### 2.5. Sample Analysis with Atomic Absorption Spectrophotometer (AAS)

Analysis of metals in both water and urine is done using an AAS. The AAS operating procedure is to turn on the AAS tool, then the standard solution and sample are added into the test tube available on the AAS device, the computer AAS device is adjusted, the AAS flame and cathode lamp are turned on, and then the standard solution is aspirated into acetylene air, indicating the resulting measurement reading must be zero by pressing the zero button. In succession, the standard solution was analyzed using an AAS and continued with a blank solution and sample solution (water and sediment). The measurement results of atomic absorption will be recorded and then calculated to get the metal concentration in the sample solution [19, 20].

### 2.6. Data Analysis

Data were analyzed descriptively and analytically. The correlation test was used to analyze the relationship between exposure of rainwater to Pb and health problems of people who use rainwater as a source of drinking water and to see the strength of the relationship carried out by looking at the value of *r* [7]. The *T*-test was used to see the difference in average between treatments, namely, analysis of differences in Pb, pH levels, and turbidity between before and after rainwater treatment with mollusk sand filtration and absorption with activated carbon.

### 2.7. Ethical Considerations

Ethical approval was obtained from the Ethics Committee for Health Research at the Makasar Ministry of Health Polytechnic (303/KEP-PTKMKS/VII). Permits were also sought from the Pontianak City government and Kubu Raya Regency before the start of the research. Participation in this study was voluntary, and informed consent was obtained from each participant at the time of the study after explaining to them the purpose of the study and how the findings would benefit them.

## 3. Results and Discussion

### 3.1. Pb Level in Rainwater

From the results of the examination of rainwater samples originating from West Kalimantan, Indonesia, with the research locations of Pontianak City and Kubu Raya, it is seen that the rainwater in these areas contain Pb. In addition, Pb in rainwater is also found in the southeastern region of Nigeria and Australia. The trigger factor for the presence of Pb in rainwater in the region comes from environmental factors such as air pollution and rainwater collection through zinc roofs [4, 5]. The results of the examination (Table 1) show that the highest Pb levels in rainwater that come into contact with the zinc roof found in the Central Siantan District of North Pontianak have an average concentration of 222 *μ*g/l and the lowest concentration is found in Limbung village in Sungai Raya District with an average of 44.6 *μ*g/l. Meanwhile, the average Pb contained in rainwater that came into contact with the zinc roof before processing was 131.7 *μ*g/l, exceeding the permitted value set by the WHO Guideline for Drinking-Water Quality Guidelines (10 *μ*g/l (8.9)). After applying mollusk sand filtration and absorption of activated carbon, the results showed that rainwater has met the requirements of drinking water, reaching a value of 0.71 *μ*g/l. The effectiveness produced for the treatment of Pb contained in rainwater is 99.47%. Statistical tests showed that there were significant differences in the level of Pb in rainwater before and after treatment (*p* ≤ 0.001).

Plumbum (Pb) in rainwater that touches zinc roofs in Pontianak and Kubu Raya districts before treatment shows that the average level is 131.7 *μ*g/l. This shows that the quality of rainwater stored directly from the zinc roof does not meet the drinking water quality requirements. Pb in rainwater is caused by the Pb layer on the zinc roof which is also soluble in acid rainwater. According to a research conducted in Australia, the Pb content is closely related to Pb which is used to coat zinc roofs [4, 21]. Besides zinc roofing used, Pb in rainwater is also caused by environmental factors because the results of research conducted in Southeast Nigeria from 2007 to 2008 in seven target cities showed a high content of plumbum (Pb). This is caused by car emissions and industrial waste [5]. Increased Pb rainwater occurs at the beginning and end of the year due to low rainfall, while in the middle of the year, it was relatively lower due to rainwater dilution. The high Pb content in rainwater is also supported by the nature of acid rain so that the Pb dissolution rate is greater [21].

Pb found in rainwater confirms that generally rainwater has relatively good (clean) quality for drinking water but has a tendency to get polluted when it is in the atmosphere and when it drops on the ground. The contamination which happens in the atmosphere can be caused by dust particles, microorganisms, and gasses such as NO_*x*_, CO_*x*_, and SO_*x*_. These pollutants are sourced from the emission of vehicles and industries and also can be sourced from the roof materials as the collector and container for rainwater [10, 11, 13]. The results of analysis from several countries such as Palestine and Australia mention that dust particles, heavy metals, and bird feces affect the physical quality of rainwater such as color and taste [22–24].

The presence of Pb in rainwater in Pontianak City is higher (211.6 *μ*g/l) compared to Pb in rainwater in Kubu Raya Regency (51.75 *μ*g/l). The fort is a rural area. This showed that the level of air pollution contributes to the presence of Pb in rainwater. Some physical, chemical, and biological air pollution parameters are quite high, for example, dust particles, CO, and heavy metals such as Pb. The emission particles are produced by vehicles and industries; especially in urban areas, Pb pollution will be greater [23–25].

There were sources of lead (Pb) in rainwater stored from a roof. Pb found in rainwater is due to dust particles in the air which has lead and stick on the roof in urban areas and can be sourced from the combustion process of fuel from vehicles and industries [12]. Lead can also be sourced from dust particle contamination as a result of land clearing by fire for farming and planting purposes and from the materials used as roof to collect and store rainwater [9, 25].

The reason why Pb concentration found in rainwater is high is not only influenced by dust particles produced by land-clearing activities or fuel combustion but also influenced by the material used for collecting and storing the rainwater [9, 25]. This can be seen from the results of examination of Pb concentration in rainwater on the zinc roof. The content of Pb found in the rainwater that came into contact with the zinc roof was higher than that found indirectly in stored rainwater, i.e., 131.7 *μ*g/*l* and 109.7 *μ*g/l. The presence of Pb in rainwater that is accommodated through zinc roofs is caused by air pollution and is also caused by corrosion of the zinc roof due to acid rain. Pb as a zinc roof coating also dissolves in rainwater.

The high Pb dissolved in rainwater is also because of natural conditions in Pontianak and Kubu Raya which are the locations in the tropics and are positioned right on the equator; therefore, the sun always passes them throughout the year. Because of these conditions, Pontianak and Kubu Raya always get full sunshine throughout the year and rain all day, especially in October and March [13, 26]. Therefore, air pollutants resulting from land-clearing and fuel combustion activities produce emissions of air pollutants such as tetraethyl lead (TEL) and tetramethyl lead [23]. Tetraethyl lead and tetramethyl lead particles will break in the air with the help of sunlight into monoethyl-Pb, diethyl-Pb, and triethyl-Pb. These three organic Pb components are easily soluble in water. Pb in rainwater is not only related to a number of factors such as sunlight, air humidity, and Pb types that are produced from the breakdown process but are also influenced by acid-base rainwater. In general, rainwater is soft water with a fairly high acid level, pH < 5. Soft water and pH < 5 will cause Pb to have high solubility and increase its concentration; therefore, when rain falls, Pb will easily dissolve in rainwater and enter rainwater storage and is unacceptable or not suitable for consumption [27].

### 3.2. Rainwater Turbidity

In Table 2, it is shown that the average turbidity of the rainwater which came into contact with the tin roof before the treatment has the highest level which is found in the subdistrict of Siantan Hulu of Pontianak for 22.26 NTU, and after the treatment, it had been lowered to 9.84 NTU. Meanwhile, the average turbidity of rainwater which came into contact with a tin roof found in 40 houses is 20.0 NTU, and after treatment, the level had been lowered to 5.67 NTU. The turbidity level after treatment of rainwater using a filtered tube has met the requirement for drinking water which is 5 NTU. The effectiveness level of turbidity reduction after treatment is 72%. The statistic test has shown a significant difference in the turbidity of rainwater before and after treatment (*p* < 0.001).

Generally, turbidity in rainwater is caused by suspended solid substances, either inorganic or organic [28, 29]. The high level of turbidity shows that rainwater has been polluted physically, chemically, and biologically. Physical pollutants included animal waste (bird) and dust particles produced by land-clearing activity by fire in rice fields and plantation. Chemical pollutants from emission produced by the combustion process of fuel from vehicles and industries included chemical-contained tin roof material used to collect and store rainwater. Meanwhile, microbiological pollutants come from viruses and bacteria found in the air. because these pollutants and Pb used as zinc roof coatings result in high turbidity of rain water and are not suitable for consumption [24, 25].

### 3.3. pH of Rain Water

It is known from the examination results in Table 3 that the lowest rainwater pH which came into contact the tin roof before treatment found in Siantan Hulu of Pontianak was an average of 4.62, and after the treatment, it had the highest escalation to 7.001; meanwhile, the average pH level of rainwater which came into contact with the tin roof before the treatment found in 40 houses was 5.16, and after treatment, it had increased to 6.95. Statistical tests showed that there were significant differences in the pH of rainwater before and after treatment (*p* ≤ 0.001).

Soft water such as rainwater with pH less than 5 will cause the level of metal solubility high, especially lead (Pb) solubility. Pb solubility comes from the piping system and other metal substances; as it is used for the roof to collect and store rainwater, it will be corrosive and will dissolve in rainwater. Therefore, the low pH of rainwater (pH < 5) will affect the solubility of poisonous metals such as Pb, making the rainwater unfit to be consumed [14]. Based on the high level of Pb, turbidity, and low pH found in rainwater, which is above the allowable value set the WHO, we need appropriate technology for the treatment.

**Figure 3:**
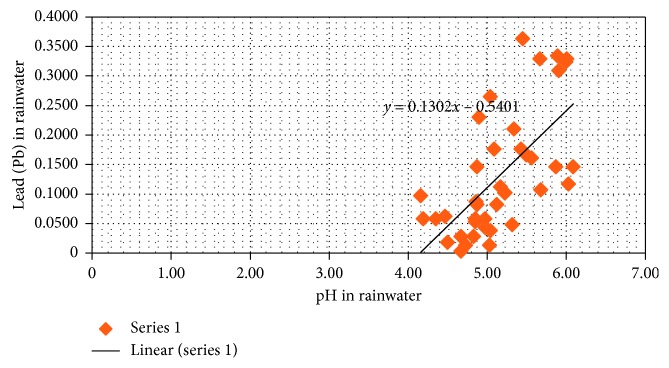
pH of rainwater to Pb content in rainwater in West Kalimantan.

Low pH in rainwater is influenced by air pollution from industries and land combustion. Air pollution is in the form of CO_x_, NO_x_, and SO_x_ which results in acid rain [30]. The gas reacts with rainwater (H_2_O) to form carbonic acid (H_2_CO_3_), sulfuric acid (H_2_SO_4_), and nitric acid (H_2_NO_3_) [31, 32]). Acidic rainwater will cause corrosiveness on the zinc roof and has a high solubility against heavy metals such as Pb [32] (Figure 3). This condition results in Pb being used as a zinc roof coating to dissolve in rainwater, and Pb particles released in the air that come from burning fuel are also dissolved in rainwater. So that in filtering rainwater before the process of absorbing activated carbon, it is necessary to increase pH to maximize the absorption process. Increasing pH is carried out using media that have alkaline compounds, such as mollusk sand which has CaCO [33] and activated carbon that has a pH 8 and 9.5. In alkaline conditions, OH ions will be larger, resulting in deprotonated mollusk sands so that metal cations are bound. After going through the Pb activated carbon media, they will be absorbed by activated carbon [34].

### 3.4. Pb in Rainwater and Public Health

Pb is a heavy metal which has the highest affinity for sulfur and attacks its bonds the enzymes. As a heavy metal, Pb is classified as a hazardous pollutant [35]. Pb is present in water in the form of Pb (OH)_2_. Pb metal is widely used in zinc coating industries and piping work. Leaded gasoline is the main source in the atmosphere and the face of the earth. Most Pb on the earth enters the natural aquatic system and accumulates, which can eventually enter the body of animals and humans. If absorbed into the human body, lead (Pb) can cause children's intelligence to decline and body's growth to hamper and can even cause paralysis. Other symptoms of Pb metal poisoning are as follows: nausea, anemia, and abdominal pain [36].

There is a tendency between the levels of exposure to Pb in rainwater with Pb levels in urine for those who consume rainwater as their drinking water. Statistical analysis has shown that there is a correlation between exposures to Pb in rainwater and public health disorders. The higher the level of exposure to Pb in rainwater, the bigger the chances for the public to suffer health disorders by consuming rainwater (*r* = 0.3).

**Figure 4:**
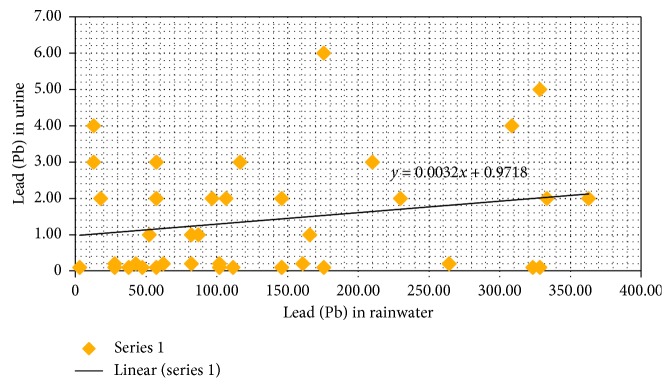
Pb levels in rainwater against Pb urine content (mean: 1.39; median: 1.00; SD: 1.54; min.: 1.00; max.: 6.00).

There is a tendency between the high level of Pb exposure in rain water and the high level of Pb in urine (health disorder) in public who consume rain water as their drinking water (Figure 4). There is also a meaningful correlation: a moderate correlation strength between the high level of Pb in rain water to the amount of Pb concentration found in urine in public health. Lead (Pb) is toxic to the human body, which is caused by food habits and consumption activities of food/drinks. Pb is not only known to be toxic but also can accumulate in the human body. Based on the results of research in Abuja, Nigeria, it is seen that the occupational exposure increases the level of lead in the blood, which consequently increases the health risk of the exposed people [37]. The exposure source and the high level of Pb concentrations are caused by toxication or health disorders among public. Research conducted in Riyadh shows that Pb concentration in domestic drinking water is higher than that in bottled drinking water and also that the concentration level of Pb in the blood of those who consumed domestic water is higher than that in those who consumed bottled drinking water [38].

Based on research in Poland, it is concluded that the road dust as a by-product of exhaust and nonexhaust emissions can be a major cause of systemic oxidative stress and multiple disorders. Substantial amounts of road dust are repeatedly suspended, in particular, at traffic signals and junctions where more braking is involved, causing a potential threat to pedestrians; especially, children HQ indices calculated for the analyzed traffic-related elements were all lower than 1.0, potentially indicating noncarcinogenic effect. The HI index for selected metals, for example, Cd, Cu, Co, and Pb, for adults fell within the safe value. However, in the case of children, the HI values exceeded the safe level of 1.0 for road dust, sludge from storm drains, and roadside topsoil in all investigated cities [39].

### 3.5. Rainwater Treatment

To decrease the content of Pb in water, especially in rainwater, we can use several methods: the timer setting for collecting and treatment by using the filtration method and absorption method using activated carbon. Filtration and absorption methods are usually used to process the ground water and underground water which have a high level of metals such as Fe, Hg, and Pb. For that reason, treatment to reduce the content of Pb in rainwater is needed. The method used to decrease the pollutant level in rainwater is using a filtration tube combined with gravel, mollusk sand, and activated carbon.

The decrease in Pb concentration and turbidity level and an increase in pH happened after the substances or materials contained in rainwater passed through a filtration tube which consisted of activated carbon coconut shell granules, mollusk sand, and gravel. The tube length was 120 cm with 20 cm thick gravel, 35 cm thick mollusk sand, and 45 cm thick activated carbon granules. Mollusk sand media are generally used with a thickness of 20 cm. However, the thicker the passive mollusk, the better it will reduce turbidity. The high level of turbidity found in rainwater is caused by dust particles including Pb particles, bird waste, and microorganisms, or it usually depends on the characteristic of pollutants in an area or a city. The mollusk sand medium filter in the tube will form a film layer that will function effectively in filtering the pollutant particles like dust, either metals such as Pb or nonmetals such as bacteria and viruses, and the color of rainwater.

The effectiveness degree of the mollusk sand filter is higher in decreasing Pb concentration and turbidity and in increasing the pH of rainwater compared to ordinary sand and activated carbon used for filtration; for example, river water treatment using quartz sand can increase pH by around 4.7% and rainwater treatment can increase pH by 2.9%, while mollusk sand can increase pH in rainwater treatment by 26%. Mollusk shell sand showed better function in increasing the rainwater pH; this happened due to CaO (94.1%), Na_2_O (1%), and SiO (1%) present in mollusk sand. Mollusk sand also absorbs heavy metals found in water and holds suspended materials, and thus mollusk sand is very useful and used as a filtration medium in water treatment [28, 29].

Shell is one of the mineral sources which come from sea creatures, and it can be been ground into small pieces and has high carbon. Minerals contained in the shell are variable and high; for example, calcium contained in the shell is 66.7%, magnesium 22.28%, and SiO_2_ 7.88%. That is why minerals found in shells which naturally have attrition or decomposition can increase rainwater pH and their carbonate content can oxidize Pb in rainwater [40].

Rainwater is not always a good clean water source that is ready to be consumed; physically, it does not have color, taste, and clarity. Rainwater is affected by the area where the rain falls. In rural areas, rainwater could be polluted by waste produced by land-clearing activities, pesticides, and animal wastes, while in urban areas, rainwater could be polluted by chemical substances dissolved in water which are tasteless and colorless and cannot be seen. A chemical substance such as Pb could come from the materials such as roofs, paint, tin, tar, dust, and asbestos. Pb could also be produced from a volcanic eruption and gas emission from fuel combustion of vehicles and industries. Rainwater contamination is not only caused by chemical substances but also can be sourced from microorganic matters such as bacteria, virus, and parasite [23, 24].

Due to the varied pollutants found in rainwater, the turbidity level has increased. The turbidity level, especially in rainwater, can also be sourced from suspended solid substances, organic and inorganic substances, and bioorganisms such as bacteria, viruses, and parasites which are airborne pollutants [24]. To decrease or reduce airborne pollutants in rainwater, filtration method by applying mollusk sand and sorption of activated carbon of coconut shells are conducted. The filtration of rainwater is done by passing the rainwater through a porous medium for particle substances which cannot be separated by sedimentation process. Pollutants that escape from filtration during rainwater treatment can be handled using absorption process; therefore, the filtration method by applying mollusk sand and absorption of activated carbon was proven to decrease the rainwater turbidity level, whose average level before the treatment was 20 NTU, and after treatment, it was 5.67 NTU with effectiveness of 72% and has met drinking water need.

Rainwater treatment using the absorption process aims to decrease suspended organic and inorganic substances which escape from the filtration process; therefore, this process functions to decrease metal substances found in rainwater such as lead (Pb). Pb concentration in rainwater which does not meet the requirement for drinking water has changed and is able to meet the requirement to be able to consume. The result of this research shows that Pb concentration in rainwater does not meet the requirement for drinking water, with the average Pb concentration found in rainwater before treatment being 131.7 *μ*g/l and after treatment decreased to 0.69 *μ*g/l with the effectiveness of 99.7%.

The ability of activated carbon to absorb Pb substances in rainwater is because activated carbon has relatively big microspore and mesopore volume, which is very much possible to absorb pollutants (including Pb) in inadequate amount. Activated carbon is one of the absorbents whose carbon atom structure is amorphous, mainly consisting of free carbon and also has a deep surface; therefore, it has a good absorption ability [41]. Therefore, to decrease the Pb concentration level and turbidity and to increase the pH of rainwater, it is best to apply the filtration process using mollusk sand in the filtering tube and also by applying absorption method of activated carbon. The result shows that the level of decreasing effectiveness for Pb is 99.47% and 72% turbidity and the pH increases, with an average of 5.16 before treatment and 6.95 after treatment and the effectiveness of pH increasing to 26%. After the treatment, the Pb concentration level went down to 0.69 *μ*g/l, turbidity level to 5.6 NTU, and pH up to 6.95. This result has met the need for drinking water quality of Pb concentration level at 10 *μ*g/l, turbidity at 5 NTU, and pH level at 6.5–8.5 [14, 42].

Sorption between activated carbon and rainwater containing Pb is strongly influenced by the pH of rainwater [27]. Absorption by activated carbon carried out at high pH tends to provide optimum results because in alkaline conditions an oxide compound of Pb element will be sorbed by the sorbent. To increase the pH in acidic rainwater, mollusk sand filtration medium is used before sorption using activated carbon, which is where mollusk sand can increase the pH of rainwater.

Backwash is a method used in the treatment of filtration devices. Backwash must be carried out continuously at regular intervals to maintain the effectiveness of the filtration device [43, 44]. The time period for the backwash has not yet been found to be explained in the literature. It has just been mentioned that the backwash indicator needs to be done. Backwash is done with the processed indicator which starts to become *cloudy*, and the effluent flow rate decreases to 30% or less from the initial value [44, 45]. Saturation of filtration media depends on the intensity of use and the load of Pb rainwater contamination. If, after being linked, backwash processed results do not show differences with raw water, then the filter media must be replaced with new media. Backwash can be active because the bond that forms on the activated carbon and Pb is an irreversible reaction. Pb absorbed by activated carbon can be released again under pressure. The current given to release Pb ions with carbon and mollusk sand must be reversed.

## 4. Practical Implications of This Study

This study found that rainwater in the tropics experienced heavy metal pollution with pollutants such as lead (Pb). Acid rain due to air pollution and the condition of the community using a zinc roof to hold rainwater aggravates the Pb level in rainwater. The Pb content in rainwater affects public health with early indications of Pb in the urine. This study also provides an alternative solution to the problem of providing clean drinking water by filtration by using mollusk sand media and activated carbon to eliminate this contamination. Other efforts can also be made by providing substitutes for Pb-zinc roofing with an environmentally friendly roof for high air polluted areas.

This study has limitations on measuring the public health impact caused by exposure of drinking water to lead as far as the Pb content is present in urine. So it is necessary to further study the impact of exposure to Pb so that it can provide further information and other efforts in controlling these impacts.

## 5. Conclusion

West Kalimantan has experienced lead pollution (Pb) in rainwater. The pollution of rainwater with heavy metals is not only caused by air pollution but also by the use of zinc roofs with Pb coatings to strengthen the bonds of zinc layers with iron layers. For purification of water contaminated with lead (Pb), a simple filtration method using mollusk sand media and activated carbon can be used. For this reason, the need to reduce exposure of rainwater to lead is carried out by replacing roofs with non-Pb-coated roofs and using filtration method as rainwater treatment before using rainwater as drinking water.

## Figures and Tables

**Table 1:** Average Pb concentration in rainwater before and after treatment.

City/regency area	Subdistrict/village	Before treatment (*μ*g/l)	After treatment (*μ*g/l)	Drinking water requirement
Pontianak	Siantan Hulu	201.3	0.75	0.01 mg/l (10 *μ*g/l)
	Siantan Tengah	222.0	0.77	
Kubu Raya	Limbung village	44.6	0.85	
	Kuala Dua village	58.9	0.47	
Mean		131.7	0.71	
Median (min.-max.)		**101.8 (3.2**–**363.0)**	**0.1 (0.1**–**3.0)**	
SD		**0.1046669**	**0.0001434**	
*P* ^a^	≤0.001^*∗*^			

Source: primary data; ^a^*T*-test (*α* = 5%); ^*∗*^significant at *p* ≤ 0.05.

**Table 2:** Average turbidity level in rainwater before and after treatment.

City/regency area	Subdistrict/village	$\bar{X}$ before treatment (NTU)	$\bar{X}$ after treatment (NTU)	Drinking water requirement
Pontianak	Siantan Hulu	22.261	9.839	5 NTU
	Siantan Tengah	21.572	7.872	
Kubu Raya	Limbung village	17.675	2.114	
	Kuala Dua village	18.502	2.831	
Mean		**20.00**	**5.67**	
Median (min.-max.)		**18.95 (15.06–26.81)**	**2.92 (0.14–14.96)**	
SD		**3.33**	**5.15**	
*P* ^a^	**≤0.001** ^*∗*^			

Source: primary data; ^a^*T*-test (*α* = 5%); ^*∗*^significant at *p* ≤ 0.05.

**Table 3:** Rainwater pH before and after treatment.

City/regency area	Subdistrict/village	pH before treatment	pH after treatment	Drinking water requirement
Pontianak	Siantan Hulu	4.626	7.001	6.5–8.5
	Siantan Tengah	4.937	6.981	
Kubu Raya	Desa Limbung	5.357	6.791	
	Desa Kuala Dua	5.721	7.014	
Mean		**5.16**	**6.95**	
Median (min.-max.)		**5.06 (4.61–6.09)**	**6.96 (6.31–7.43)**	
Sd		**0.52**	**0.21**	
*P* ^a^	**≤0.001** ^*∗*^			

Source: primary data; ^a^*T*-test (*α* = 5%); ^*∗*^significant at *p* ≤ 0.05.

## Data Availability

The data used to support the findings of this study are available from the corresponding author upon request.
